# Active and Healthy Confinement: Care Recommendations on Activity, Sleep and Relationships

**DOI:** 10.3390/healthcare11121773

**Published:** 2023-06-15

**Authors:** Alexandra González Aguña, Marta Fernández Batalla, Sara Herrero Jaén, Andrea Sierra Ortega, María Lourdes Martínez Muñoz, José María Santamaría García

**Affiliations:** 1 Intensive Care Unit, Henares University Hospital, Foundation for Biomedical Research and Innovation (FIIB HUIS HHEN), 28822 Madrid, Spain; 2 Research Group MISKC, Department of Computer Science, University of Alcala, Polytechnic Building, University Campus, Barcelona Road Km. 33.6, 28805 Madrid, Spain; marta.fdezbatalla@gmail.com (M.F.B.); sara_herrerojaen@yahoo.es (S.H.J.); andreeasierra@gmail.com (A.S.O.);; 3 Torres de la Alameda Health Centre, Community of Madrid Health Service (SERMAS), 28813 Madrid, Spain; 4 Mejorada del Campo Health Centre, Community of Madrid Health Service (SERMAS), 28880 Madrid, Spain; 5 Meco Health Centre, Community of Madrid Health Service (SERMAS), 28880 Madrid, Spain; 6 Care Management, Community of Madrid Health Service (SERMAS), 28020 Madrid, Spain

**Keywords:** counselling, COVID-19, exercise, interpersonal relations, lifestyle, nursing care, sleep

## Abstract

Persons that lived through periods of confinement suffered an impact on their physical and mental health. The adaptation of the lifestyle in relation to activity, sleep and social relationships is key to facing these periods of confinement. The aim is to validate a series of care recommendations aimed at being able to maintain an active and healthy confinement, which serves to prepare the population for future health crises. This study is part of a general strategy based on a care recommendation guide for COVID-19. The validation was carried out by a group of experts using the Delphi technique through a questionnaire that uses the Content Validity Index (CVI) and considers high validation those with a score >0.80. A total of 75 care recommendations are proposed: 30 on activity–exercise (CVI = 0.82), 14 on sleep–rest (CVI = 0.83) and 31 on roles–relationships (CVI = 0.83). Additionally, 49 recommendations achieve high validation. The care recommendations integrate a person-centred model, which addresses individual characteristics (age, health status, professional role). An active and healthy confinement requires respecting social distance measures, maintaining a balance between physical activity and sleep, and using technologies to promote social contact, which promote well-being and avoid depression and anxiety.

## 1. Introduction

The International Year of the Nurse and Midwife coincided with the COVID-19 pandemic. This global crisis limited the actions planned for the Nursing Now campaign but made visible the added value for the society of nurses and their ability to lead health policies [1,2,3,4,5,6,7]. Currently, the World Health Organization has declared the end of the global state of emergency, but the experience lived should serve to learn and prepare us for similar health situations that require isolation of people [8].

This global health process was a crisis in many aspects but, at the same time, a unique opportunity for health care research: a new situation, with a temporally and geographically delimited beginning, with isolation measures, quarantines, a period of confinement in many regions, expansion of the use of technologies and information systems that allowed all kinds of data to be shared [9,10,11,12]. The most restrictive measure was confinement (home isolation, quarantine, lockdowns) because it had a significant impact on lifestyle behaviours and patient care anywhere in the world [13].

Three functional patterns stand out for their direct relationship with these strict measures: activity–exercise pattern, sleep–rest pattern and roles–relationships pattern. The confinement had a drastic impact on the amount and type of physical activity (bodily movement performed by skeletal muscles that demand energy expenditure) and in sleep habits (resting state in which the body is not active, and the mind is unconscious) [14,15,16]. They are two closely related functional patterns because they influence each other [15,17]. At the same time, the interruption in lifestyles produced isolation and weakened social relationships [18,19]. Social connections are an inherent need of people. The absence of meaningful connections produces loneliness, and people can experience social isolation. While social interaction produces benefits in physical and psychological health, the de-crease in social contact and the interruption of lifestyles are risk factors for deterioration of emotional and mental health [20,21,22,23,24,25]. Researchers conducted numerous studies, and journals published all kinds of articles on this topic. The topics of the articles include how nurses face the health emergency and the measures taken in the health services [26,27,28,29,30,31,32]. Other studies describe the situation in vulnerable groups of pregnant women, lactating women or the elderly [33,34,35]. However, these publications did not include any nursing theory or standardized nursing language until mid-2020, when the first articles identifying care diagnoses in NANDA-I taxonomy appeared [36,37,38,39]. In Spain, one of the countries most affected by COVID-19 and with the strictest conditions of confinement for several months, a group of nursing researchers applied an analysis based on professional nursing care and the Taxonomic Triangulation technique on this document [37,40,41].

The same researchers promoted in Madrid the project “*Recommendations for care in the face of the COVID-19 pandemic for all healthcare settings*”, a project to face the possibility of a future with a chronic risk of infection from COVID-19 or similar health problems [42]. Thus, recommendations are *“statements designed to help end-users make informed decisions on whether, when and how to undertake specific actions such as clinical interventions, diagnostic tests or public health measures, with the aim of achieving the best possible individual or collective health outcomes*” [43]. These recommendations reach the population as health advice in the form of brief, opportunistic (taking advantage of professional meetings) interventions that include information based on evidence and that motivate behaviour change [44]. The recommendations are classified in Marjory Gordon’s Functional Patterns [45].

### 1.1. Theorical Framework

The conceptual model of the project is the *Knowledge Model about Person Care*, a person-centred model. This model is centred on the person, designed from the individual person’s perspective as well as those of their families and communities, all of whom benefit from health services. Caring attention focuses on needs with an integrated approach. The vulnerability of the person and the risk of the environment determine the predisposition to suffer care problems [46,47,48]. The limitations of knowledge, skills and motivation act on this predisposition and lead the person to a potential situation of suffering care problems. These problems may be in the subclinical phase or manifest with a wide range of severity. The result of these care situations will influence the level of health, which will later be part of the basal situation of the person [42].

This conceptual model assumes the following nursing theories: Orem’s Self-Care Theory for the relationship between care demands and the capacity for self-care; the Neuman Systems Model because COVID-19 acts as a stressor that attacks the individual system and can alter the integrity until it affects the basic core [49].

Furthermore, the recommendations are classified in Marjory Gordon’s Functional Patterns [45]. This classification establishes eleven functional patterns, of which three are used in this study:

- Activity–exercise pattern: motor skills, daily autonomy, leisure…
- Sleep–rest pattern: sleep habits, perception of rest, relaxation, drugs…
- Roles–relationships pattern: structure and roles in the family, work and community, responsibilities, personal satisfaction…

In this sense, this research provides a novel vision because it integrates a conceptual and professional model of nursing care compared to previous publications that studied the impact of COVID19 for specific areas and from the intervention in the acute moment of isolation. Previous published research left a gap to offer a comprehensive and integrated recommendation guide based on a person-centred model in any possible situation. In addition, the purpose is that the drafting of the recommendations has a specific format (with a specific focus of care interest) but is flexible—that is, it allows the nursing professional to individualize the recommendation for each person they care for.

### 1.2. Main Aim

The main aim of the study is to create a guide of validated care recommendations against COVID-19 in functional patterns directly related to confinement: activity–exercise, sleep–rest, and roles–relationships.

## 2. Materials and Methods

This study is part of a larger project entitled “*Recommendations for care in the face of the COVID-19 pandemic for all healthcare settings*”, led by the Care Management of the Madrid Health Service (SERMAS), and which sought to develop and validate of a care recommendation guide with integrated and comprehensive guidance.

### 2.1. Design

The design followed a mixed method. This methodology combines quantitative and qualitative research. Therefore, the design included bibliographic review techniques, elicitation with experts and consensus for validation. The analysis involved numerical scores and possible qualitative individual assessments.

The study methods were compliant with the Good Reporting of a Mixed Methods Study (GRAMMS) [50]. The GRAMMS framework consists of six aspects to evaluate the use of mixed methods and includes questions such as the justification for using this method, the description of the design, the sample, data collection, analysis or limitations.

### 2.2. Participants

The study included three groups of participants with different profiles and purposes for the procedure. The different groups are summarized below:

- Coordinating group: Group of principal researchers who led the study, designed the files, selected the participants, held the meetings and collected the data from the experts. The group had four experts, the majority of whom were women (75%) aged between 33 and 63 years (mean 44.75), which reflects the global population of nursing professionals in Spain. All the experts had more than ten years of professional experience, with profiles of management, teaching and, in addition, a high academic degree (three with a PhD degree and one with a master’s degree).
- Expert group for technical validation of the data collection tool (questionnaire): The coordinating group recruited a group of clinical experts through intentional sampling to represent care professionals and health centres from the entire Madrid region. A group of five clinicians external to the research validated the table proposal and accompanying explanation sent to each expert for data collection. The profile of this group was clinical nurses with experience in research based on consensus by a group of experts. The result of its validation were recommendations aimed at clarity and explanation about the content and about what the participants are asked to do. The purpose was to ensure that each table could be completed as a self-administered questionnaire.
- Expert panel: The group consisted of five nurses (two worked in an adult hospital, one in a children’s hospital, one in family and community care, and one in out-of-hospital emergencies) and a physiotherapist. All were women with at least ten years of professional experience and who had participated in at least one nursing research project; four had a postgraduate degree, and three had teaching experience. The physiotherapist was included because in Spain this profession depends on nursing directors, and they work together with nursing in rehabilitation. None of the participants represents a company or scientific society related to health or pharmaceuticals to avoid possible conflicts of interest.

Each participant only participated in one group—that is, no individual played two different roles in the research.

### 2.3. Procedure

The procedure was divided into two phases that combine different research techniques to reduce the limitations that appear when only one method is used. This mixed methodology made it possible to combine already published scientific evidence, the personal experience of different professionals and validation through a representative group of different professionals who provide care in the health system. Thus, this combination of techniques reduces the limitations that appear when only one method is used.

#### 2.3.1. Phase I—Acquisition of Expert Knowledge

##### Research Study Period

The acquisition of knowledge through elicitation with a group of experts took place from May 2020 to June 2021.

##### Data Collection Tool

The data collection tables used the Marjory Gordon Functional Patterns classification broken down into elements to guide the assessment. This classification is used in the Madrid Primary Care medical records to assess the person and their environment. The files were integrated into the Microsoft 365 Excel^®^ tool.

The design of each table was dedicated to a specific functional pattern and included a column with the different elements of the pattern, followed by cells so that the participants of the group of experts could record their contributions with their corresponding reference.

The activity–exercise pattern included the following elements: physical exercise, functional capacity, dependency, physical limitations, work and leisure activities and technologies. The sleep–rest pattern included the following elements: sleep pattern, environmental factors and drugs. The role–relationship pattern included the following elements as a guide: family, social support, labour relations, safe environment and violence.

The external group validated the data collection tool—that is, the questionnaire to be self-administered in the validation phase of the recommendations. None of these experts participated in the other phases of the study.

##### Presentation Session and Knowledge Acquisition

The field phase of the research began with a presentation session. The coordinating group met with potential participants to present the study. The session lasted 90 min and included a theoretical presentation (objective, methodology, expected results) and a time for questions and doubts. All participants were informed and agreed to participate in the project.

Subsequently, the participants received an email with all the summarized information on the study, and subsequently, the coordinating group sent each functional pattern with a time interval (two weeks) for each participant to respond individually, reflectively, and anonymously, justifying each contribution with references, which were clinical guidelines, government websites or published research. Once each table was completed, the participant sent his response to the coordinating group, who sent him the following functional pattern.

The coordinating group brought together all the contributions and unified the recommendations based on their content. The contributions with the same main axis (equivalence of word or meaning) were unified in a single recommendation to which all the bibliographical references provided by the experts were linked. Each recommendation includes the percentage of participants who provided at least one bibliographical reference. This list of care recommendations was used in the next phase of the study.

#### 2.3.2. Phase II—Validation of Recommendations

##### Research Study Period

The second phase aimed to validate the care recommendations designed from the information obtained in the previous phase and ran from July 2021 to December 2021.

##### Evaluation Questionnaire

The coordinating group sent the result of the unification, the proposed recommendations, to the group of experts. Each participant received a file with a similar structure to the previous one to complete a self-administered questionnaire. The questionnaire consisted of a table with the unified recommendations of each pattern along with the percentage of participants who had provided a record to build that recommendation.

The question that guided the evaluation was “Do you consider this recommendation adequate for the care of people at risk or diagnosed with COVID-19?” The evaluation used a 5-point Likert scale, where 1 means “not representative” and 5 means “totally representative”, and a section so that each participant could express in natural language any consideration that she wanted. This methodology was similar to similar studies with outcome indicators from the NOC taxonomy [42,51].

##### Validation Analysis

The analysis used the content validation index (CVI), which is obtained by dividing the number of positive responses (scores of 4 or 5 points) by the total number of responders. Non-responders are not included. Similar investigations establish the cut-off at different scores, so that for the present study, it is decided to establish 0.50 for approved, with recommendations with >0.80 points being “Approved with high validation”. Recommendations with <0.50 were considered not validated [52,53,54].

The coordinating group returned the global result (as a group) of the CVI score to each participant so that each participant could revalidate or modify their level of agreement that they expressed in the first phase.

The data were acquired and validated by the same group of experts in both phases of the procedure: the first phase was individual, anonymous and based on publications, while the second phase was collective, by consensus and adapted to the clinical realities observed by the professionals.

### 2.4. Ethics

The study did not involve obtaining health data from individuals or population groups because it was based on published scientific evidence. The study also did not use information on health institutions, groups of specific health professionals or on care processes that are related to any institution. In this sense, the study does not imply any ethical consideration of special relevance.

However, all the participants received complete information on the study before their participation, all agreed to participate without obtaining any benefit for it, allowed the use of the data collected in the questionnaires on personal profile and validation of recommendations, and furthermore, none presented any conflict of interest. The Care Management, as the reference care area of the Madrid Health Service (SERMAS), approved the study design and monitored its performance.

## 3. Results

The global project obtains 624 contributions from the participants, which are unified in 258 care recommendations. The three patterns analysed in the study obtain 178 records that the coordinating group unifies into 75 care recommendations. These recommendations are analysed below according to functional pattern.

### 3.1. Functional Pattern: Activity–Exercise

The activity–exercise functional pattern includes 74 records that are unified into 30 recommendations. The mean CVI is 0.82 (from 0.33 to 1.00). The participants gave a high validation to 18 recommendations (60.00% of total recommendations for this pattern) and did not validate one (3.33%). The tables contained two blank responses because two participants did not score one item each.

The three recommendations with the highest score address care actions to maintain an active lifestyle for the general population (adapted to their functional capacity), for the elderly with multicomponent programs and for people in the process of rehabilitation after the COVID-19 disease. Many of the approved recommendations detail the characteristics of exercise according to the population group (children, the elderly, groups with health disorders prior to the pandemic), according to the type of activity (aerobic, flexibility, balance) and the way of exercising (frequency, time, rest). Other recommendations include technologies as a means to promote and facilitate physical activity in periods of confinement. Finally, a recommendation includes the use of a mask because the pandemic situation still advised its widespread use. This recommendation must be contextualized at the time of the pandemic and the situation of each person.

The only unapproved recommendation is “Use active video games.” This recommendation does not specify a group or situation, and the evidence of its use is still scarce without an expert or trainer to avoid inappropriate use and risks.

The summary of care recommendations for the activity–exercise pattern is in Table 1.

### 3.2. Functional Pattern: Sleep–Rest

The sleep-rest functional pattern includes 33 records that are unified into 14 recommendations. The mean CVI score is 0.83 (from 0.50 to 1.00). All recommendations were validated. The participants gave a high validation to 9 recommendations (64.29% of total recommendations for this pattern). The tables contained two blank responses because one participant did not rate two recommendations.

The recommendations with the highest score are to promote sleep quality through relaxation techniques and stress reduction and, in addition, the advice to expose yourself to the sun and reduce the time in front of screens. Many recommendations are related to other functional patterns of care (food, exercise, social relationships) and with the environment (pleasant surroundings, clean and disinfected housing). In addition, the recommendations include health education and control of the use of drugs for sleep.

Care recommendations for the sleep–rest pattern are in Table 2.

### 3.3. Functional Pattern: Roles–Relationships

The roles–relationships functional pattern reaches 71 records that are unified in 31 recommendations. This functional pattern is the one that achieves the most recommendations. The mean CVI score is 0.83 (from 0.50 to 1.00). The participants gave a high validation to 22 recommendations (60.00% of total recommendations for this pattern) and did not validate one (3.23%). The tables contained two blank responses because one participant did not rate two recommendations.

The recommendations include a variety of foci of interest. The recommendations with the highest score are about keeping distance to prevent contagion, promoting technologies to maintain ties with other people, planning work times to balance loads, ensuring risk prevention for women in situations of gender violence and sending the message that people can leave each other during confinement when there is a dangerous or emergency situation. The unapproved recommendation is related to the use of masks in the general population and professionals. In this sense, the health situation changed during the study and, consequently, the experts modified their score because its use was no longer mandatory and systematic.

The summary of care recommendations for the role–relationship pattern is shown in Table 3.

## 4. Discussion

This study is part of a broader project of care recommendations for people at risk or diagnosed with COVID-19 [42]. At the end of the study, no publications were found with an aim such as that proposed in this research or with an approach based on nursing care models. However, multiple studies addressed lifestyles related to the three areas of care analysed and most closely affected by quarantine or confinement measures.

### 4.1. Functional Pattern: Activity–Exercise

Physical activity and exercise are an object of interest in studies. A search for articles related to this functional pattern during COVID-19 manages to identify a large number of investigations, with different interest groups, types of exercise and methodologies, including a systematic review [40,55,56]. The WHO also disseminated physical activity recommendations to combat the time locked up at home [57].

In general, all the studies highlight the importance of maintaining physical activity and exercise as a lifestyle and, especially, during periods of confinement because they improve physical and mental health. Physical and social activity are a modifiable factor that impacts health and mental health throughout life [58].

Health advice should consider the age, basal condition, and health situation of each person. Additionally, the indication of exercise should specify the appropriate type of activity. In general, aerobic exercise is recommended (walking, activities on static machines, climbing stairs), but strength, flexibility and even balance exercises are also important in older people. Once the exercise has been chosen, the professional must specify the frequency (daily or number of times a week), the duration in minutes (from 20–30 min to more than an hour) and the intensity (light to intense). The frequency, duration and intensity should be assessed according to the type of exercise and the person. Likewise, it is recommended to combine exercises and create multicomponent programs [40,55,56,59,60,61].

In the child and adolescent population, physical activity should be daily due to their vital stage and the benefits they achieve. During COVID-19, studies show a reduction in physical activity in the child and adolescent population [62,63]. Children and adolescents who perform physical activity show better levels of life satisfaction, affection and self-regulation compared to the same inactive population [64,65]. Some research analysed the impact of strategies to maintain physical exercise in students during confinement through online courses [66,67]. The elderly population must adjust the activity, frequency, duration and intensity to their physical condition with aerobic and balance exercises, which can be done without specialized equipment. Benefits include prevention of falls and aspiration pneumonia, increased duration of antibody levels, and overall improvement in health outcomes [56].

In this sense, the proposed care recommendations reflect the results of these similar investigations because they highlight the importance of physical activity as a lifestyle. The main recommendation is to avoid inactivity when physical space is limited during confinement. In addition, the validated care recommendations include general aspects about different types of exercises (flexibility, joint mobility, ergonomics), specific by age group (from childhood, adults to the elderly) and different health situations (people with and without disease, with and without a previous health history that limits exercise), and in addition, the care recommendations integrate the use of technologies to promote exercise.

### 4.2. Functional Pattern: Sleep–Rest

Regarding sleep–rest, there are two relationships that reflect the impact received. On the one hand, the change of life to a situation of confinement and concern about the world crisis affect sleep, and on the other hand, sleep habits can be modified due to the reciprocal influence with physical activity, which is also affected during the pandemic [16,17]. Thus, this functional pattern is also the object of interest for many studies (recommendations have also been published by international institutions), and frequently, the analysis of sleep habits is related to physical activity [68,69,70,71,72].

In general, research shows a deterioration in sleep during confinement in several countries, although the results differ depending on the type of population [23,25,73]. Children and adolescents in school increased the number of hours of sleep due to the relaxation at scheduled times. Adults went to bed later, woke up later and took more naps, but the feeling of quality of sleep and night rest was lower [74]. Differences in sleep habits should analyse inter-individual differences and the person’s situation such as sex, age, family role, resilience or employment. Two vulnerable populations specifically for this study are frontline healthcare workers and people with COVID-19 because items show a higher percentage of sleep disturbance [71]. Moreover, sleep is necessary to achieve rest and for periods of recovery from illnesses. Sleep disturbance is associated with worry, a feeling of social isolation, feelings of anxiety or depression [74,75,76,77].

In this sense, the care recommendations also highlight maintaining sleep habits as essential. These sleep habits depend on characteristics such as age, family role or work role. Furthermore, the recommendations proposed in this study also relate this sleep pattern to other patterns of physical activity and eating or even consider whether the person has other health problems that affect sleep (for example, the person uses a CPAP machine for sleeping).

### 4.3. Functional Pattern: Role–Relationship

Finally, the functional role–relationship pattern is closely linked to lifestyle modifications during global lockdown and quarantine periods. Social distance policies have caused negative effects on the psychosocial health of the population and, especially, in vulnerable groups with social isolation or loneliness [78,79].

In this sense, the restriction in face-to-face social relationships and the change in roles (family, work) during the pandemic have had an impact on health. During 2020 and in subsequent years, the numbers of people with anxiety, depression and other mental disorders that affect self-esteem have increased and even reached the extreme of increasing suicidal ideation [19,80,81,82,83,84,85]. Parallel to the other functional patterns analysed, the impact on individual roles, relationships and psychosocial health results depends on the characteristics of the person, their situation prior to the pandemic and the context in which they are located. For example, frontline workers and COVID-19 patients are also the most affected by processes such as depression or anxiety [81,86,87].

In this sense, the care recommendations for the role–relationships functional pattern were focused on safety issues because COVID19 affected people’s roles: family role, work role, educational role. People must attend to role maintenance and personal satisfaction to prevent mental health problems. Mental health has been the main impact in the medium-long term, and even after the COVID19 emergency has ended, the consequences for mental health have continued to grow.

Finally, both the research results and previous publications highlight the role of technologies to help maintain lifestyles but adapted to new situations. Technologies have served to carry out care practices at home such as physical exercise, aerobics, yoga, meditation, relaxation guides for sleeping, video calls with family and friends or social calls to promote the feeling of social support.

### 4.4. Limitations

The study has limitations that must be exposed to contextualize the research and facilitate its replication and translation. First, the study began in 2020 at the end of the first wave of the pandemic, when scientific evidence was still scarce and, furthermore, vaccines had not been approved. In this sense, there is currently more knowledge about the process of this disease and its treatment; there are vaccines for the population and the WHO has recently declared the end of the international emergency.

The methodology was based on evidence from sources such as guides, articles or institutional websites at a time when there was scarce scientific evidence and research with a high level of evidence had not yet been carried out. The evidence is reduced to expert consensus and literature reviews, as occurs in this study. Some recent publications include systematic reviews on specific aspects of care (physical activity, sleep) in the face of COVID-19 and confinement. These publications show the variability in the impact of COVID-19 in these care areas and, in addition, the variability in care advice (example: type, frequency, duration and intensity of exercise according to age, sex, previous condition, situation of health). Additionally, the number and profile of the expert group participants is limited and specific. The methodological quality would be higher with a larger sample and including other health professionals.

In addition, this guide to care recommendations is prepared for the regional context of Madrid, although the participants provide an overview from all nursing clinical perspectives and support each recommendation with international publications. The guideline is limited in its practical application because the results have limited external validation, and adaptation studies are necessary to apply these recommendations to other countries and health systems.

On the contrary, a strength of the study is that it compiles and synthesizes the available evidence to design a guide that serves as a tool for nursing professionals because it is designed from a conceptual nursing approach with a person-centred care model in any context. The proposed care recommendations could be used in the future for their application to other infectious processes of similar aetiology. Care recommendations could promote specific research studies on one or several aspects and apply methodologies that increase the level of evidence.

### 4.5. Relevance to Clinical Practice

Nursing is the discipline of caring for people and, therefore, should lead clinical decision-making in this professional field. This approach has already been highlighted in the Nursing Now strategy and is in the 2030 Agenda action plan [3,87,88].

The validated care recommendations represent a common knowledge base for all professionals who do research on care related to physical activity, sleep and social relationships. In this sense, the study integrates a common disciplinary model based on *Knowledge Model about Person Care*, which can be used by other researchers and, thus, increase the evidence of the recommendations. In the clinical care field, nursing professionals can use this guide of recommendations to develop personalized care advice according to the vulnerability conditions of the person and the risk of the environment. In this sense, the recommendations are flexible because they seek to be as general as possible but, at the same time, allow their adaptation to each health situation.

## 5. Conclusions

The research achieved the objective of providing a guide to active lifestyle recommendations for a healthy confinement. Active and healthy confinement requires a series of care measures that are reflected in recommendations designed from a person-centred model.

*Knowledge Model about Person Care* makes it possible to design a guide of general recommendations and, specifically, associated with three key aspects of lifestyle during periods of confinement: physical activity, sleep and social relationships. In addition, this same model allows us to analyse the available evidence, demonstrating its concordance because it integrates a perspective centred on the person, characterized by their vulnerability (age, baseline health status, family system, work role) and under the risk analysis of different contexts (confinement, first-line health workers, homes with infected people).

In conclusion, the care recommendations of this study are aimed at maintaining a healthy lifestyle during periods of confinement and are designed for the general population but allow for individual adaptation according to the life situation of each person.

## Figures and Tables

**Table 1:** Care recommendations for functional pattern activity–exercise.

Recommendations for Functional Pattern Activity–Exercise	Contributing Participants	CVI
Avoid physical inactivity; promote exercise adapted to each person and their functional capacity with progression as tolerated.	83%	1.00
For people with respiratory sequelae from COVID-19: perform aerobic exercise to recover previous basal capacity and improve psychological condition. Techniques to improve ventilation and drainage of secretions can be applied to promote improvement. Sports activity should be resumed after 7–10 days with mild-moderate intensity.	50%	1.00
For the elderly: perform multi-component programs that include aerobic, strength, flexibility and balance exercises.	50%	1.00
Identify situations limiting physical activity: chest pain, shortness of breath, arrhythmia episode, loss of consciousness during exercise, symptoms suggestive of COVI-19, vomiting, diarrhea, asthma, acute bone injuries.	33%	1.00
For people with moderate-severe severity: limit physical activity to a minimum and remain in the prone position for alternate periods.	17%	1.00
Maintain mental and physical activity to regulate emotions and body.	17%	1.00
For dependent people: maintain the therapeutic regimen of physical activity.	17%	1.00
For people in a critical situation: encourage early mobilization individually adapted to each situation, level of consciousness and collaboration.	17%	1.00
Perform flexibility exercises.	17%	1.00
For vulnerable people (children, the elderly, and a history of cardiovascular or respiratory disease): seek health advice from health care providers.	17%	1.00
For people with dementia: maintain physical activity by dancing, walking, going up and down stairs.	17%	1.00
For people with severe musculoskeletal or cardiopulmonary disorders: perform low-intensity activity.	17%	1.00
For children: respect social distancing in group activities depending on the regulations.	17%	1.00
Use technologies to perform physical exercise (aerobics, pilates, yoga, stretching)	33%	0.83
Perform joint mobility exercises.	17%	0.83
For people who are physically active outdoors: adapt protection measures to the regulations, using a mask if necessary.	17%	0.83
Maintain ergonomics during work activity.	17%	0.83
Take active breaks during teleworking of 5 min every 2 h at least.	17%	0.83
Take active breaks every 20 min, looking up about 50–60 cm.	33%	0.80
For physiotherapy professionals: apply respiratory physiotherapy techniques, body position, drainage of secretions, early rehabilitation breathing exercises.	67%	0.67
Change the mask frequently during physical exercise.	17%	0.67
For children and adolescents: encourage physical activity for at least one hour a day.	17%	0.67
For people who practice sports: adapt spaces and practice to prevention regulations.	17%	0.67
For children: reduce time in front of screens (television, mobile,) up to 1 h/day (under 4 years) or 2 h/day (from 4 years) maximum.	17%	0.67
Define level of physical activity to balance the quality of life during the period of confinement.	17%	0.67
Perform household chores manually to stay active.	17%	0.67
Follow the recommendations of the sports health services to guarantee the proper practice of sport and competition.	17%	0.67
Promote the use of technologies for monitoring health services.	17%	0.60
For people undergoing cardiac rehabilitation: implement remote therapy at home when face-to-face intervention at the health center is not possible.	17%	0.50
Play active video games.	17%	0.33

**Table 2:** Care recommendations for functional pattern sleep–rest.

Recommendations for Functional Pattern Sleep–Rest	Contributing Participants	CVI
Maintain wake-sleep routines and avoid naps or naps that last no more than twenty minutes.	67%	1.00
Avoid the use of hypnotics, do not indicate as the first treatment option.	50%	1.00
Promote the quality of sleep with relaxation techniques and stress reduction.	33%	1.00
Maintain one hour of exposure to daylight and reduce the use of screens (television, computer, tablet, mobile), especially before bed.	33%	1.00
Control the time and type of news about COVID-19 to be informed but avoid stress.	50%	0.83
Practice daily physical exercise (avoiding the three hours before going to sleep) that includes physical and mental relaxation exercises before going to sleep and reflection, breathing and stretching upon awakening.	33%	0.83
Provide a pleasant environment for rest (quiet, dark, temperature).	17%	0.83
Avoid large meals or take stimulants (caffeine, alcohol, tobacco) before bed.	17%	0.83
Know the possible adverse reactions to the drugs used for COVID-19 infection.	17%	0.83
For people using positive pressure devices (CPAP, BiPAP): use an exclusive room for sleeping, keep disposable equipment and supplies in proper condition.	33%	0.80
Promote health education on the importance of sleep.	17%	0.80
For health professionals: promote coping and resilience strategies, providing adequate material and organization of services.	33%	0.67
Keep the house clean, disinfected and ventilated.	17%	0.67
Eat foods that improve serotonin and melatonin levels at dinner.	17%	0.50

**Table 3:** Care recommendations for functional pattern roles–relationships.

Recommendations for Functional Pattern Roles–Relationships	Contributing Participants	CVI
Encourage the use of technological devices to maintain contact with family and friends.	67%	1.00
For people in a situation of gender violence: remember the telephone number 016 to request help and consult any situation (it does not leave a telephone record). In a dangerous situation, pharmacies activate the gender violence protocol when someone requests “Mask 19”.	67%	1.00
Reduce the transmission of infection within the home through self-isolation of people with COVID-19, quarantine of people at risk by contact, and masks in shared spaces.	50%	1.00
Plan work tasks to adapt them to current regulations on prevention and to distribute workloads, with defined performance roles and access to human resources support.	50%	1.00
For people in a situation of danger or emergency: Know that you can leave your home (even in a period of confinement) to go to police, judicial or other resources, without entailing a penalty.	50%	1.00
Maintain family daily routines and establish new healthy habits.	33%	1.00
Participate in local support networks and community activities.	33%	1.00
Apply measures to prevent the spread of COVID-19: social distance, disinfection, avoid closed spaces with poor ventilation.	33%	1.00
Understand dysphoric emotional reactions (anxiety, worry, hopelessness, uncertainty, irritability, etc.) and identify if they persist over time to request help.	17%	1.00
For children with periods of isolation and quarantine: Identify the risk of fear, anxiety or mental health problems arising from the interruption of their lifestyle, reduce inequality (resources, technological skills) for learning from home and help to families with less income to mitigate the negative effects of the pandemic.	17%	1.00
For people in the formative period: maintain through information and communication technologies (ICT) the follow-up of classes and relationships with classmates.	17%	1.00
Notify family and friends of the state of health, calling the emergency service in case of worsening.	50%	0.83
Use the time together to do leisure activities (conversations, photos, games, movies) for which you barely have time in daily life.	17%	0.83
Allow the participation of all family members in decisions, taking into account the opinion of minors	17%	0.83
Avoid making grandparents responsible for caring for grandchildren.	17%	0.83
Avoid visits from non-living people.	17%	0.83
Promote family adaptation to the COVID-19 crisis through reducing the emotional impact on parents, strengthen capacities to face threats, provide a structured family environment (with healthy lifestyle habits and control of uncertainty), encourage the expression of feelings of family members, act and develop skills in the face of the emotional impact on children.	17%	0.83
Share emotions and experiences, as well as participate in community actions such as letters of support.	17%	0.83
Prevent the risk of cyberviolence through family training and dissemination of messages of support.	17%	0.83
Resolve conflicts without physical or emotional violence.	17%	0.83
For children at risk of abuse: promote teleconsultation to monitor and identify risk situations, maintain home visits with personal protective equipment, administer health questionnaires and detect child abuse when they return to school.	17%	0.83
For people at risk of domestic violence: recommend the use of technologies to maintain socio-sanitary contact, promote safety during the period of confinement and disseminate codes (key words, gestures) to notify in risk situations.	17%	0.83
Ensure privacy, consent and image security in sexting practices.	17%	0.80
Know the importance of mental health (reduce anxiety, improve coping) and mental telehealth services.	17%	0.67
For people with some health process: participating in associations and groups of patients benefits the maintenance of physical care actions and helps coping.	17%	0.67
Identify the risk of falls at home to adapt the home and activities to the person.	17%	0.67
Support disadvantaged families with economic and material resources, psychosocial support and coping strategies.	17%	0.67
Promote social awareness through the media to prevent gender violence and facilitate the reporting of situations of violence.	17%	0.67
For people with work activity: adapt qualifications to market changes.	17%	0.60
Encourage adaptation to new forms of team communication, verbal and non-verbal.	17%	0.50
Recommend surgical mask to the general population and health professionals not exposed to aerosol generating procedures (AGP), and the FFP2 or higher type mask only for health professionals before AGP.	17%	0.33

## Data Availability

The data that support the findings of this study are available on request from the corresponding author. The data are not publicly available due to privacy or ethical restrictions.
